# Evaluation of myocardial performance by serial speckle tracking echocardiography in diagnosis and follow-up of a patient with eosinophilic myocarditis

**DOI:** 10.1186/s44156-022-00013-6

**Published:** 2023-01-26

**Authors:** Mohammadbagher Sharifkazemi, Gholamreza Rezaian, Mehrzad Lotfi

**Affiliations:** 1 Department of Cardiology, Nemazee Hospital, Shiraz University of Medical Sciences, Nemazee Square, Shiraz, 71936-13311 Iran; 2 Department of Radiology, Shahid Faghihi Hospital, Shiraz University of Medical Sciences, Shiraz, Iran

**Keywords:** Myocarditis, Eosinophilia, Echocardiography, Ventricular dysfunction, Left

## Abstract

**Background:**

Speckle tracking echocardiography (STE) has been used as an adjunct diagnostic modality in patients with eosinophilic myocarditis. Its serial dynamic nature, however, has never been reported before.

**Case presentation:**

A 17-year-old boy presented in cardiogenic shock state. His full blood count revealed an absolute eosinophilic count of 11.18 × 10^3^/μL. An emergency 2D echocardiogram (2DE) showed global left ventricular hypokinesia with LVEF = 9.0% by Simpson’s method and a large amount of pericardial effusion. STE showed a global longitudinal strain (GLS) of − 4.1%. Because of his poor clinical status and presence of marked hypereosinophilia and the possibility of eosinophilic myocarditis (EM), parenteral pulse therapy with methylprednisolone and inotropes was started with subsequent improvement within the next 48 h. Over the next few days, he had his first cardiovascular magnetic resonance imaging (CMR), which showed late gadolinium enhancement (LGE) in different cardiac regions. After two weeks of therapy, he left the hospital in a stable condition, with LVEF = 38.0%, and GLS = − 13.9%. He did well during his two months of outpatient follow-ups and was found to have an absolute eosinophil count of 0.0% on several occasions. Unfortunately, he was re-admitted because of treatment non-compliance with almost the same, albeit milder, symptoms. The WBC count was 18.1 × 10^3^ per microliter, and the eosinophilic count was 5.04 × 10^3^/μL (28%). Heart failure treatment and high-dose prednisolone were started. After 15 days of admission, he got better and was discharged. During both hospital admissions and several months of follow-up, he had multiple 2DEs, STE, and two CMR studies. None of his STEs were identical to the prior studies and were dynamic with frequent wax and wanes throughout the admissions and follow-ups. Thus a single admission-time STE study was not sufficient enough to properly predict the patient’s outcome. Follow-up STEs showed new sites of myocardial involvement despite the absence of eosinophilia.

**Conclusion:**

The use of STE in this patient, proved to have an added value in the evaluation and stratification of the left ventricular function in patients with EM and can be used as a diagnostic adjunct to CMR for diagnosis of EM.

## Background

The chronic presence of high eosinophil cells in the serum (hyper-eosinophilia), for any reason including immunological, allergic, or infectious reasons, can result in cardiac tissue damage. This damage is caused by eosinophil-derived neurotoxins, proteins, and reactive oxygen species, especially in the form of myocarditis, known as acute eosinophilic myocarditis (EM) [1, 2]. The clinical manifestations of EM are mainly non-specific and overlap with other cardiac diseases, ranging from asymptomatic to dyspnea, chest pain, dysrhythmias, cardiogenic shock, syncope, and sudden death varying based on the extent of cardiac damage [3].

Endomyocardial biopsy, the gold standard diagnostic method, is an invasive method and not always applicable because of poor clinical conditions in the patient, lack of facilities, and experience [4]. Hyper-eosinophilia is also undetectable in about 24% of the patients because of eosinophil deposition in tissues [5, 6]. Despite several diagnostic methods suggested, neither changes in the electrocardiogram, such as ST–T segment abnormalities, nor the wide range of non-specific features in two-dimensional echocardiogram (2DE), are able to distinguish EM from other cardiac diseases. However, gadolinium-based cardiovascular magnetic resonance (CMR) imaging is recommended as an accurate non-invasive diagnostic tool for acute myocarditis and EM [7, 8]. It can show tissue abnormalities (such as necrosis, fibrosis, edema, and hyperemia), as well as changes in wall thickness, chamber dimensions, ventricular function, and amount of pericardial effusion. However, CMR is not always accessible, and its interpretation requires specific training and experience. Therefore, looking for an alternative tool for early EM diagnosis is a medical necessity [9].

Speckle tracking echocardiography (STE) is a sensitive tool for early detection of myocardial involvement and left ventricular (LV) dysfunction using strain and strain rate, especially in patients with acute myocarditis [9–11]. Nevertheless, the role of STE in early diagnosis, as well as the role of serial STEs to assess their evolutionary changes over time in patients with EM and comparison with their concomitantly performed 2DEs and three CMRs studies, has never been reported before. Herein, we present such a novel case.

## Case presentation

**Fig. 1:**
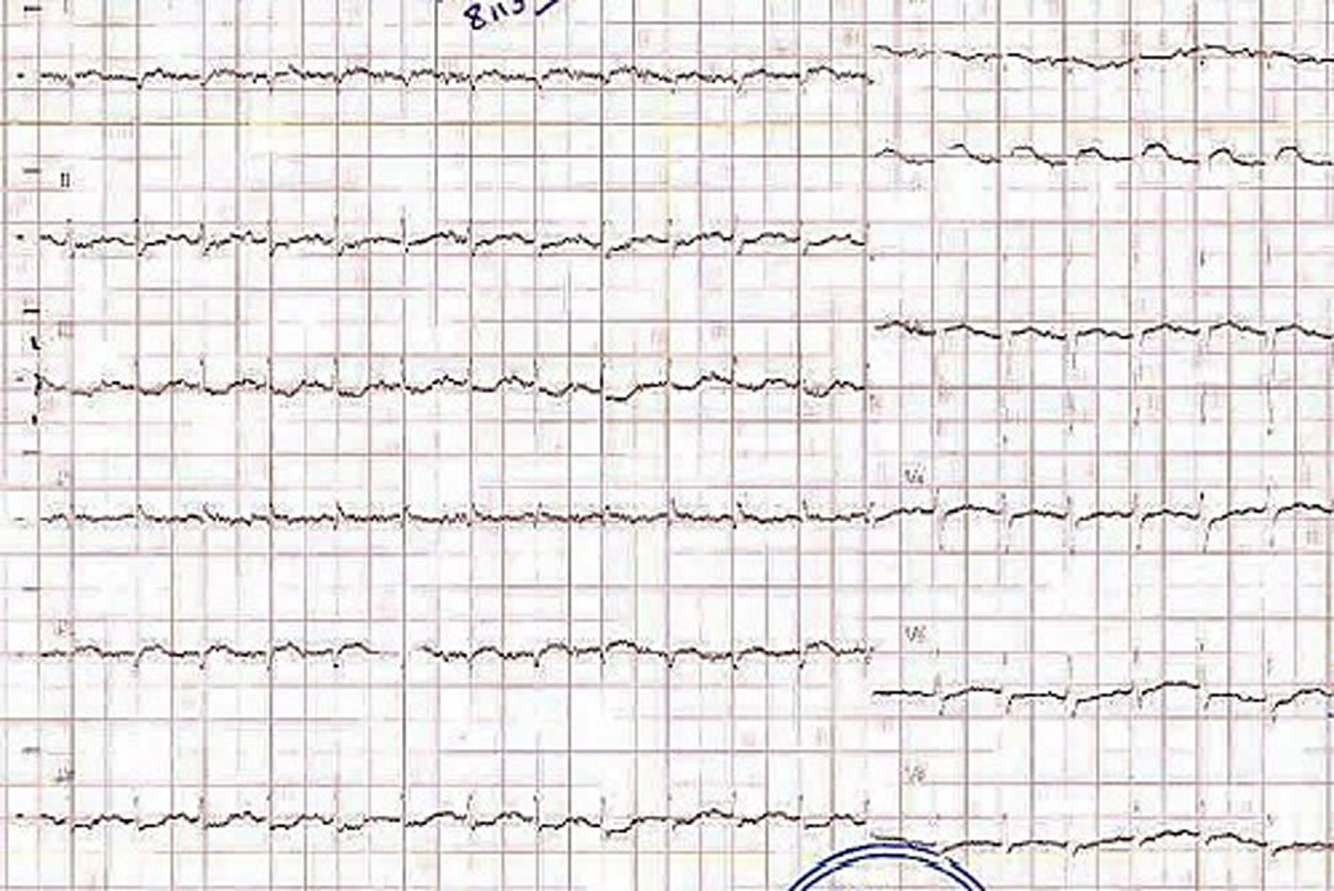
The first electrocardiogram showing low voltage QRS complexes, sinus tachycardia plus non-specific ST–T changes

**Fig. 2:**
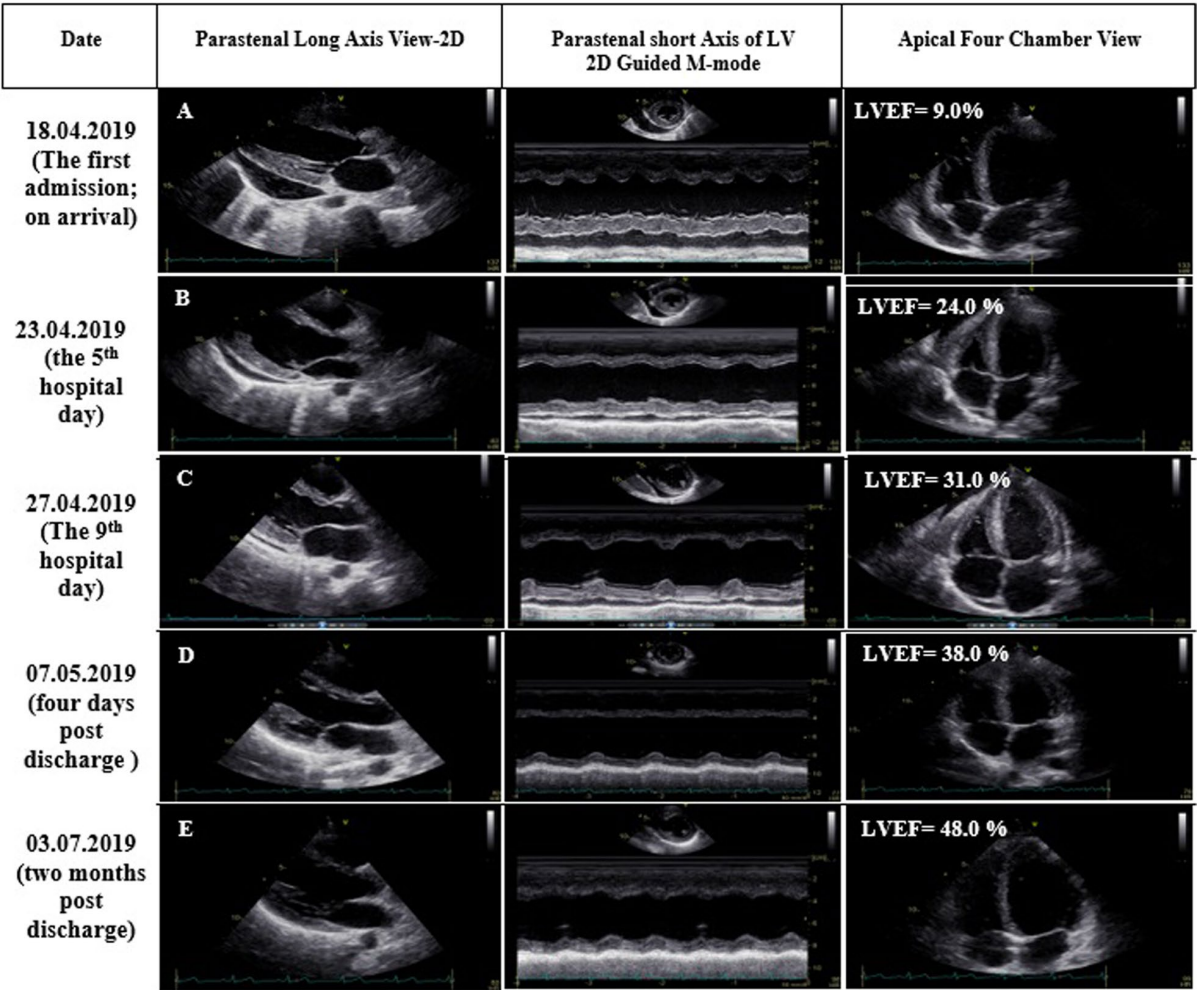

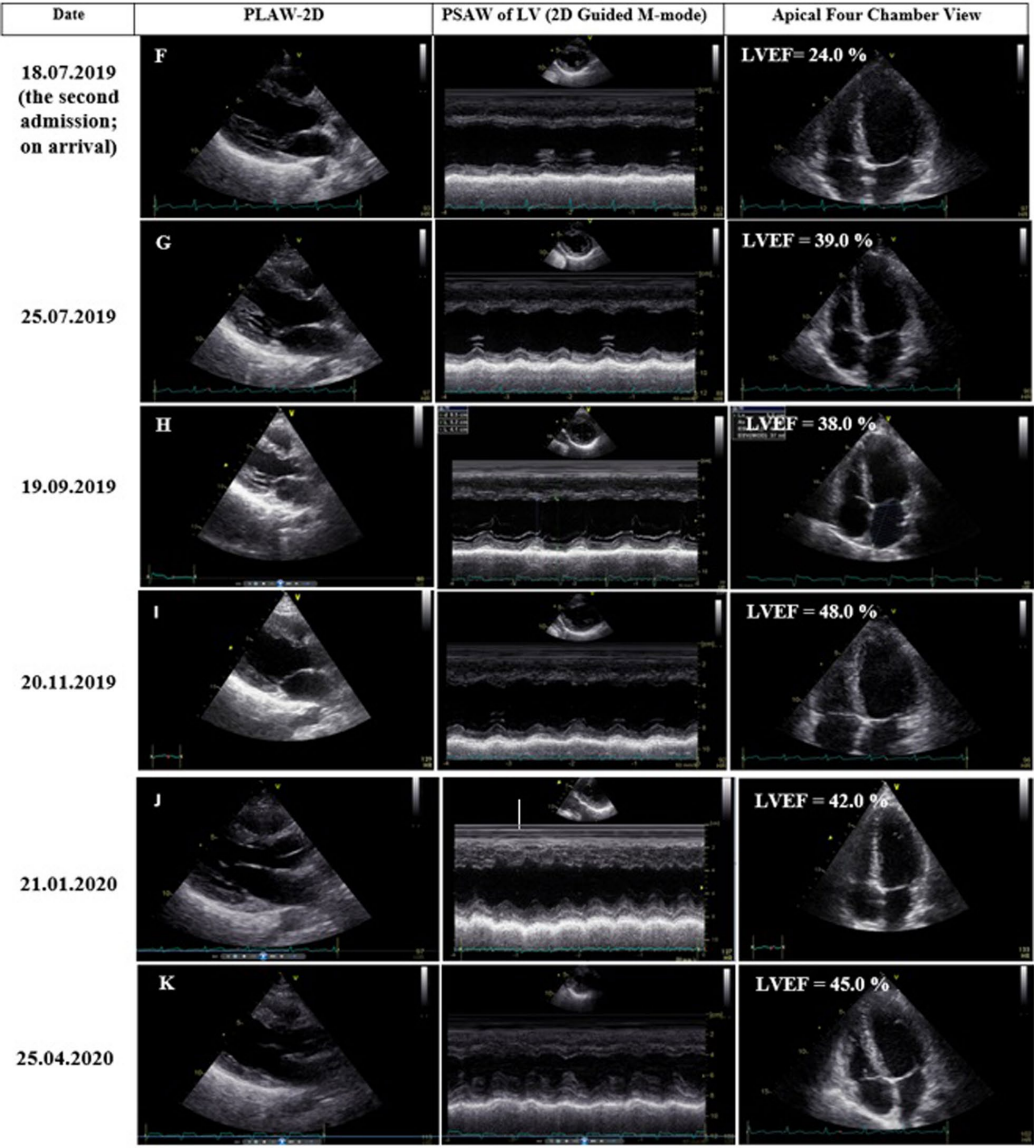
Serial transthoracic echocardiographic studies. **A**–**E** views: first admission and later follow-ups. On arrival, there was a large amount of pericardial effusion in addition to left ventricular systolic dysfunction. During the hospital course and then after, there were improvements in the amount of pericardial effusion, as well as thickness and systolic function of the myocardium. **F–K** views: second admission and later follow-ups. On arrival, there was systolic dysfunction in addition to scanty thinning of the ventricular myocardium. Later in and out of the hospital, there were improvements in the myocardial thickness, as well as the systolic function of the myocardium

A 17-year-old boy with asthma was referred to the emergency department in cardiogenic shock. He had experienced high-grade fever, dyspnea, productive cough, and profuse sweating for the past ten days. On arrival, he appeared pale, quite anxious, and in severe respiratory distress. The systolic blood pressure was 70 mmHg (by the manual measurement using a sphygmomanometer), the respiratory rate was 34/min, his pulse was weak but rapid (about 130/min). The body temperature was 39.2 °C. The heart sounds were muffled with audible pericardial friction rubs and bilateral harsh breathing sounds. His full blood count revealed a total white blood cell count of 23 × 10^3^/μL and an absolute eosinophilic count of 11.18 × 10^3^/μL. The sinus tachycardia and wide-spread non-specific ST segment and T wave changes were evident on his electrocardiogram (Fig. 1). His chest X-ray showed cardiomegaly and pulmonary congestion. The 2DE revealed increased myocardial thickness with global LV hypokinesia and an ejection fraction (EF) of 9.0% by Simpson’s method. Large pericardial effusion was evident, too (without tamponade). The right ventricle (RV) size and systolic function were normal (Fig. 2A). The inferior vena cava appeared plethoric and non-compressible.

**Fig. 3:**
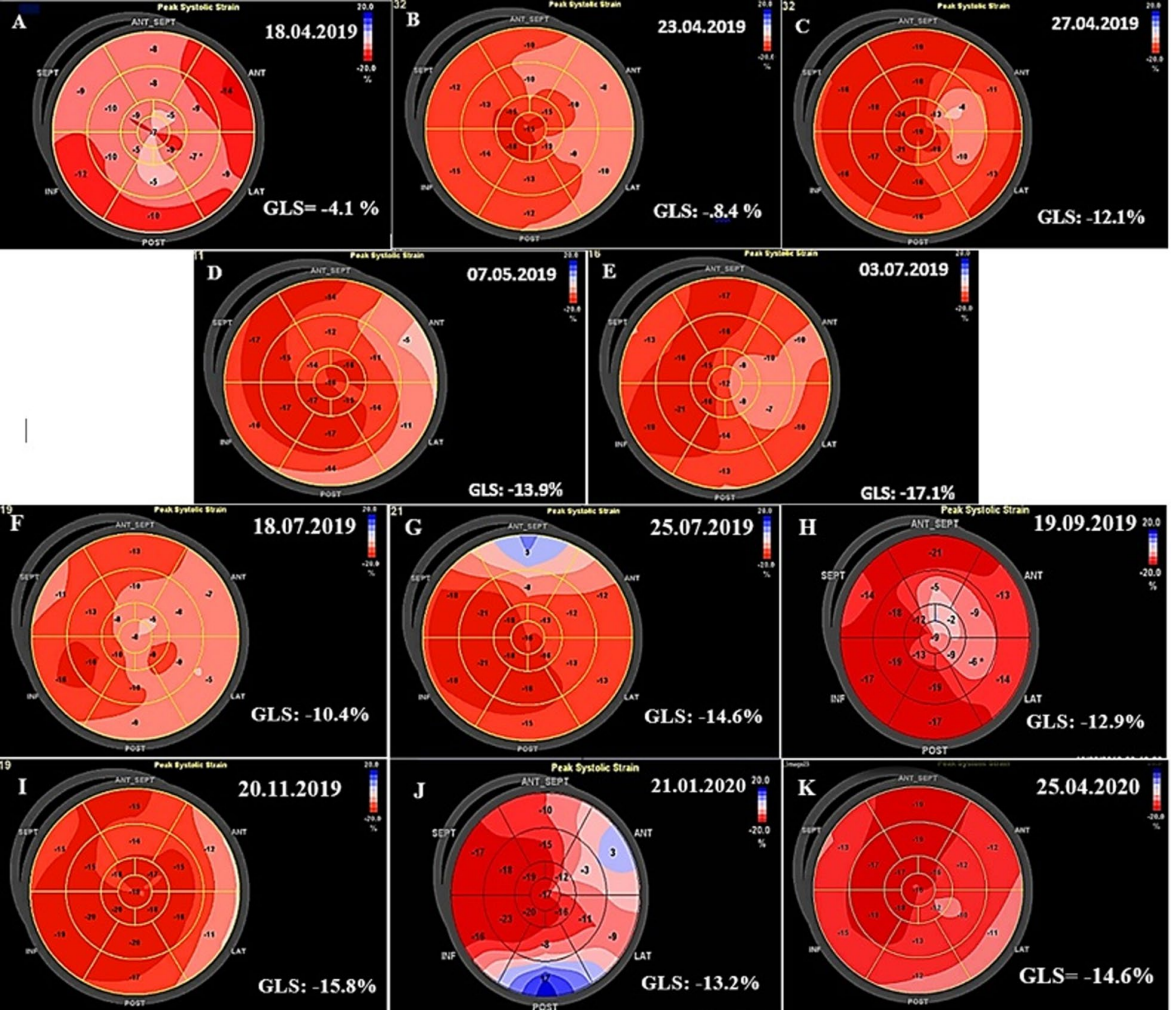
**A**–**K** Global longitudinal strain measurements at different intervals. The frequent wax and wanes show the severity of reduced longitudinal strain and the changing cardiac involvement sites both throughout first and second admissions. Anterior (or Antero-lateral*), Anteroseptal (or Anterior*), Inferior (or Infero-septal*), Lateral (or Infero-lateral*), Posterior (or Inferior*), Septal (or Antero-Septal*) (*) According to segment definitions by Voigt and co-workers [12]

Due to the unavailability of CMR at the admitting centre, STE was performed using a commercially available standard ultrasound scanner (E9, General Electric Medical Systems, Horten, Norway) with a 2.5 MHz transducer. All the images were obtained with a frame rate of 50–70 frames/s. Left ventricular longitudinal peak systolic strain (LS) was assessed using standard apical views. Strain and strain rate measurements were performed offline with dedicated automated software (EchoPAC PC, version 6.0.0, GE Healthcare, Chalfont St. Giles, United Kingdom). This showed extensive reduced longitudinal strain (LS) in nearly all segments with a global longitudinal strain (GLS) of − 4.1% (Fig. 3A) [12].

Endomyocardial biopsy was deferred by the patient and his parents because of his very poor clinical condition. However, the presence of severe global LV dysfunction and hyper-eosinophilia raised the possibility of EM and warranted initiation of immediate therapy without having a definitive diagnosis.

Therefore, pulse therapy with 1000 mg intravenous methylprednisolone, inotropes, and intravenous heparin was immediately started in the intensive care unit. After adequate hemodynamic improvement, low-dose beta-blockers and angiotensin-converting enzyme inhibitors were given and gradually up titrated to 6.25 mg carvedilol twice daily and 12.5 mg captopril twice daily. Furosemide 20 mg IV was given as needed to correct pulmonary congestion.

**Fig. 4:**
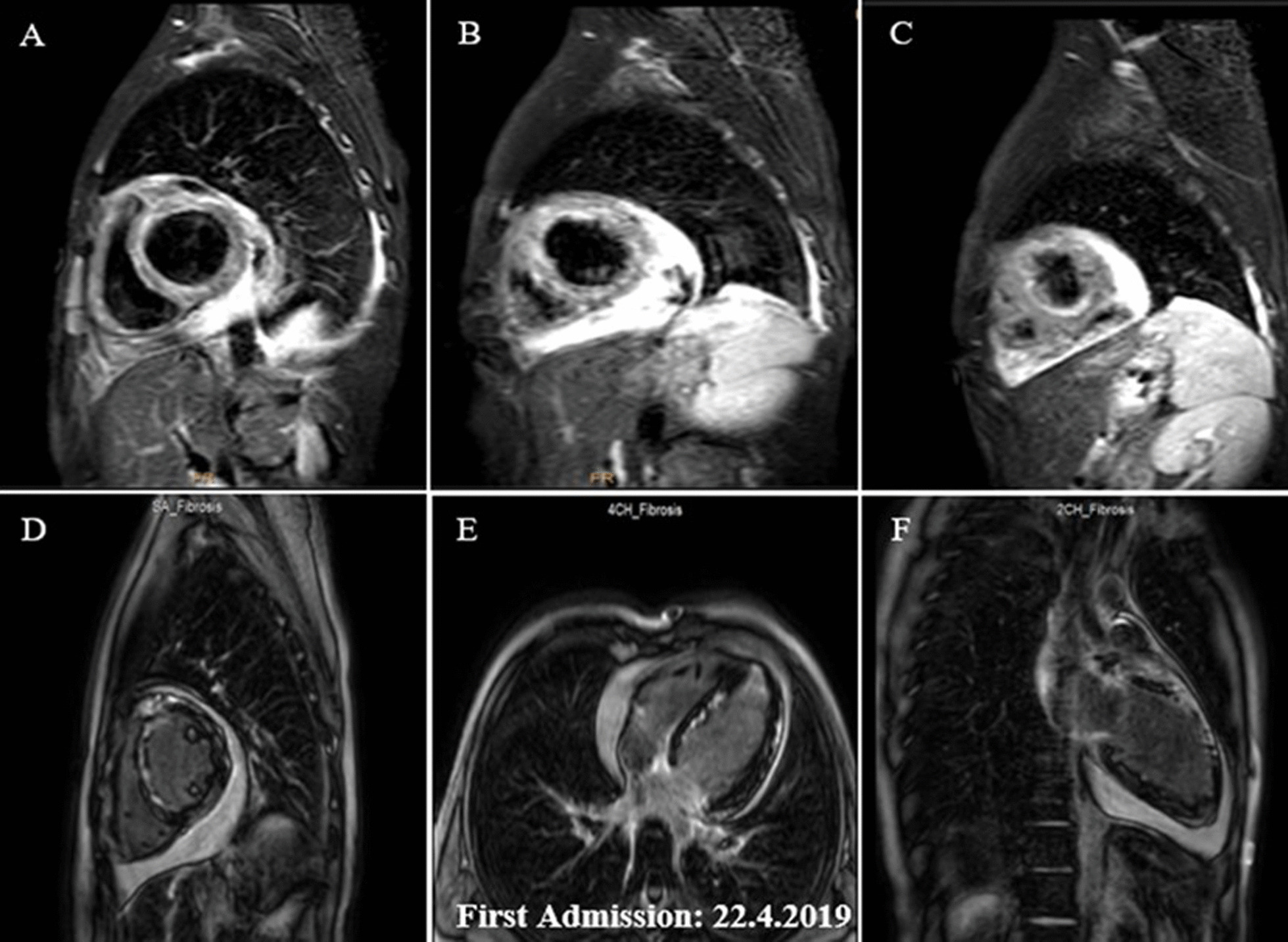
**A–F**. Short axis basal, mid and apical fat saturated T2 weighted (STIR) images demonstrating mainly subendocardial and with lesser degree midmyocardial and epicardial edema at different basal, mid and apical walls (**A**–**C**). Short axis, 4-chamber and vertical 2-chamber post contrast views showing subendocardial, midmyocardial and epicardial LGE in different basal, mid and apical walls, consistent with acute inflammation (**D**–**F**). Papillary muscle involvement is shown in **D**

He became afebrile within the next 48 h, and his respiratory distress dramatically improved. The eosinophil count reached less than 1000/µL, and following hematological consult, it was decided not to do bone marrow aspiration/biopsy at that time. His general condition, as well as 2DE and STE findings, improved further over the next few days (Figs. 2B, 3B). So that he could be transferred to another well-equipped center for CMR evaluation, which showed significant improvement in the LVEF (37.0%), reduction of the pericardial effusion, subendocardial and epicardial edema, and late gadolinium enhancement (LGE) in different cardiac walls (Fig. 4A–F). His inotropes were stopped, and his heart failure medications were adjusted to even lower doses, and after two weeks of hospitalization, he was stable enough to go home on low-dose prednisolone (7.5 mg daily). The pre-discharge echocardiography showed a remarkable reduction in LV dimensions and pericardial effusion. The LVEF was 31.0% (Fig. 2C), and STE showed a GLS of − 12.1% (Fig. 3C). Also, 2DE and STE results four days after discharge revealed LVEF of 38.0% (Fig. 2D) and GLS of − 13.9% (Fig. 3D). In the outpatient follow-up, significant clinical improvement was observed with zero eosinophil count on several occasions while receiving supportive therapy. His two-month post-discharge 2DE showed an LVEF of 48.0% (Fig. 2E) with GLS of − 17.1% on STE (Fig. 3E).

**Fig. 5:**
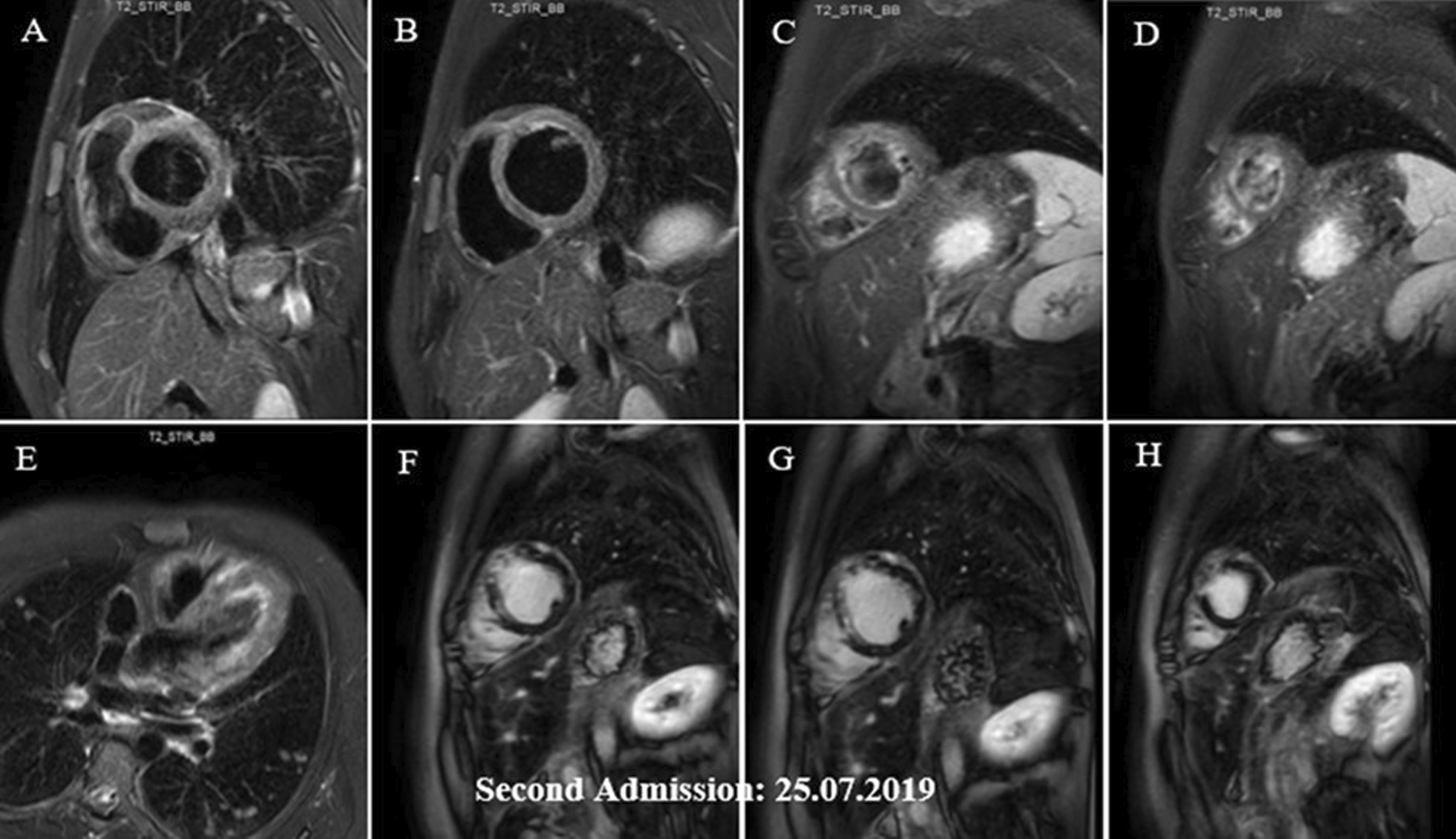
**A–H** Short axis basal, mid, apical and 4-chamber STIR images demonstrating subendocardial and with lesser degree midmyocardial and epicardial edema in different LV walls (mainly the apex) and also in RV walls (**A**–**E**), which has decreased compared with previous study (shown in Fig. 4**A**–**C**), indicative of partial improvement. Short axis post contrast views showing subendocardial, midmyocardial and epicardial LGE in anterior, septal and with lesser degree anterolateral LV walls, mainly consistent with residual inflammation and with lesser degree areas of midmyocardial scar formation in anterior and septal walls (**F**–**H**)

**Fig. 6:**
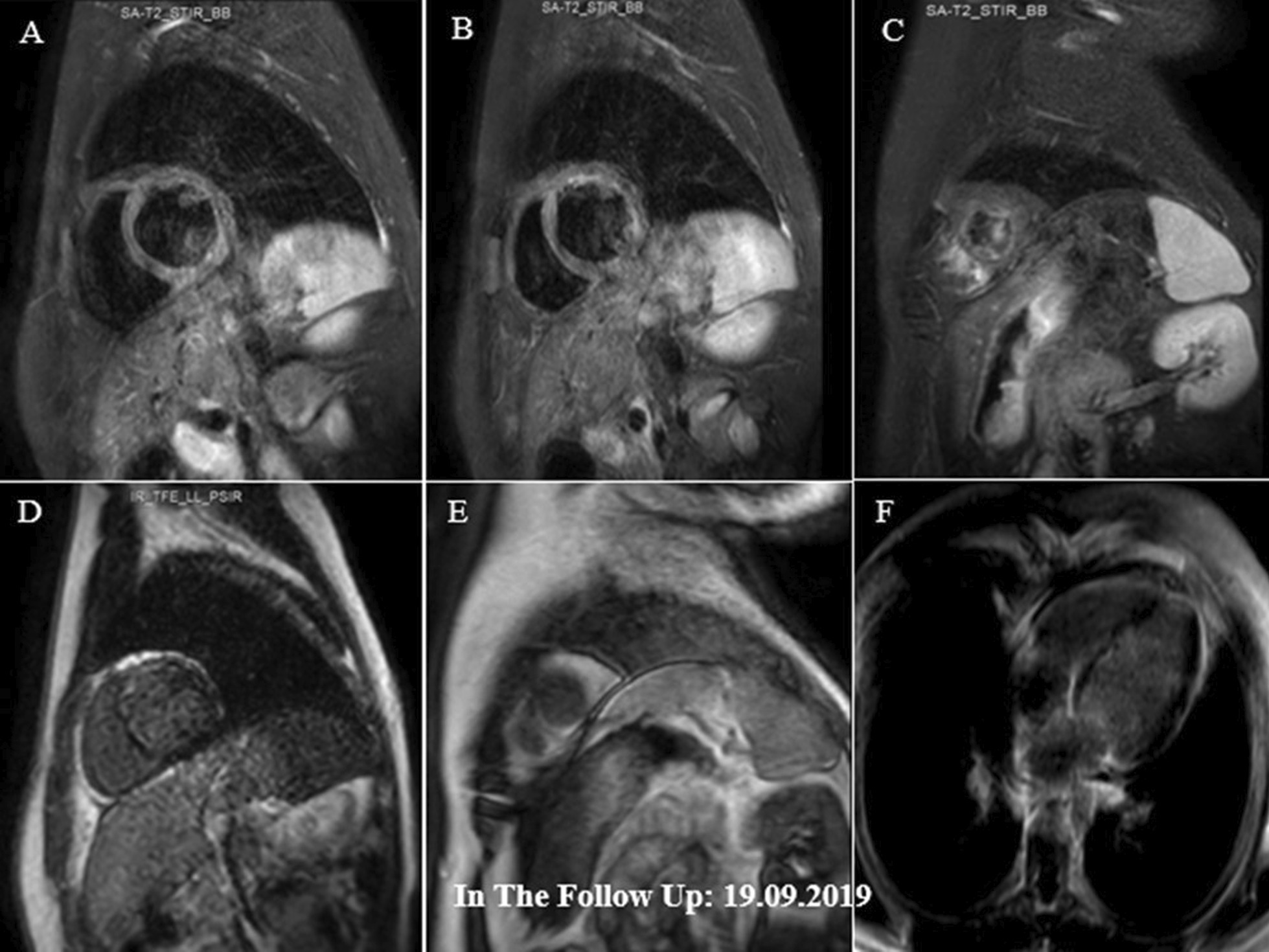
**A–F** Short axis basal, mid and apical STIR images demonstrating subendocardial edema in different mid and apical walls (mainly in the apex) and also in RV apical region (**A**–**C**). These have been decreased compared with previous study (shown in Fig. 5**A**–**E**), suggestive of significant partial improvement. Short axis and 4-chamber post contrast views showing subendocardial LGE in anterior and septal LV walls, consistent with small areas of residual inflammation and scar formation (**D**–**F**)

About two weeks later, however, he required re-admission because of non-compliance with treatment. The symptoms were almost the same as his first hospital admission, albeit milder: a body temperature of 38.2 °C, respiratory rate of 28/min, pulse rate of 112/min, and blood pressure of 95/80 mmHg. An emergency 2DE and STE showed minimal residual pericardial effusion and drop in LVEF (24.0%) with new areas of reduced LS and a GLS of − 10.4% (Figs. 2F, 3F). The white blood cell count was 18.1 × 10^3^/µL with an eosinophilic count of 5.04 × 10^3^/µL (28.0%). High-dose oral prednisolone (60 mg per day) was immediately started. The recovery, however, was slow, and by the 4th day, he became afebrile, and his respiration improved. The left ventricular wall thickness was decreased in his 2DE (Fig. 2G), and there seemed to be evidence of scar formation in the basal anteroseptal region in his STE (Fig. 3G). The CMR showed some LGE in the anterior LV wall and evidence of mild mid-myocardial scar formation in the anteroseptal region (Fig. 5A–H). After 15 days of admission, he left the hospital in a medically good condition. Six weeks post-discharge, concomitant 2DE, STE, and CMR showed LVEF of 38.0% (Fig. 2H), GLS of − 12.9% (Fig. 3H), and evidence of significant improvement in subendocardial edema at different mid and apical LV segments, in addition to subendocardial LGE in the anterior and septal LV walls, consistent with small areas of scar formation (Fig. 6A–F). This was, however, in contrast to the STE that did not show any significant reduced LS to raise the possibility of scar formation. Within the next several months, he did well, and seven months after his first admission, while receiving 12.5 mg of prednisolone and with a 0.0% absolute eosinophil count, his LVEF was found to be 48.0% by Simpson’s method (Fig. 2I) and no evidence of significant reduced LS (scar formation) was seen in his STE (Fig. 3I). These strange and unexpected wax and wanes in his STE were seen once again in his next evaluation (Date: 21.01.2020), which revealed scar in the base of anterior and posterior walls and reduced LS in the midsegments of the anterolateral wall and base to mid part of the lateral wall (Fig. 3J). Some degree of recovery in these areas, however, was noted in his last study on 25.04.2020 (Fig. 3K). A brief timeline of the patient’s clinical presentations and paraclinical findings of both admissions is shown in Table 1.

**Table 1:** The timeline of the patient’s clinical presentations and paraclinical findings during both admissions and follow-up period

Date	Day	Clinical description	Eosinophilia	STE (GLS) (%)	Bull’s eye (W&W)	LVEF by 2DE (%)	CMR
18.04.2019 (first admission)	1	Fever, dyspnea, hypotension	Significantly positive	− 4.1	Reduced LS (all segments)	9.0	
23.04.2019	3–5	No more fever, less dyspnea	Getting better, None on the 5th day	− 8.4	Present	24.0	Acute inflammation (severe LGE)
27.04.2019	9	Doing much better	Absent	− 12.1	Present	31.0	
07.05.2019	19	Doing well	Absent	− 13.9	Present	38	
03.07.2019	76	No symptoms	Absent	− 17.1	Present	48.0	
Treatment non-compliance leading to disease recurrence							
18.07.2019 (second admission)	91	Fever, dyspnea	Present but less than the first admission	− 10.4	Reduced LS mainly in the base, mid and apical regions	24.0	
25.07.2019	98	Feeling much better	Absent	− 14.6	Present	39	Subendocardial and epicardial edema
19.09.2019	153	Doing well	Absent	− 12.9	Present	38.0	Small areas of residual inflammation and scar formation
20.11.2019	216	No symptoms	Absent	− 15.8	Present	48.0	
21.01.2020	278	No symptoms	Absent	− 13.2	Present	42.0	
25.04.2020	373	No symptoms	Absent	− 14.6	Present	45.0	

*CMR* cardiac magnetic resonance, *GLS* global longitudinal strain, *LS* longitudinal strain, *LVEF* left ventricular ejection fraction, *STE* speckle tracking echocardiography, *2DE* two-dimensional echocardiography, *W&W* wax and wanes

## Discussion and conclusions

Since there are very few case reports of acute EM, reporting new cases could add to the literature [8]. The following points were the most interesting findings in this patient:

The myocardial performance, as measured by GLS, had dynamic changes, what we called as wax and wanes, throughout both admissions and follow-up periods (Fig. 3A–E, F–K). These changes were associated with the appearance, followed by partial or total disappearance and occasional reappearance of new STE findings with varying degrees of severity and new myocardial wall or segmental involvement. For example, areas that initially had severe reduced LS and seemed to represent ‘scar formation’ were subsequently proved to be false and mostly disappeared later on without us having any explanation.

Sometimes the follow-up STE studies showed new sites of myocardial involvement despite the absence of eosinophilia.

In their review of 179 patients with EM, Brambatti et al. [5] reported a median age of 41 years (interquartile range: 27–53 years). This patient, however, was much younger (17 years old), emphasizing the necessity of considering the possibility of EM in adolescents and even in children with appropriate disease presentations.

The combination of clinical symptoms, hyper-eosinophilia, 2DE findings, including severe LV systolic dysfunction and low EF, drove the physician’s suspicion towards EM. Because of the unavailability of CMR in the admitting hospital and refusal of the patient and his parents to have an endomyocardial biopsy, STE was performed as an alternative, available non-invasive procedure to help early cardiac diagnostic assessment and treatment of the patient. In fact, prompt steroid and supportive therapy could be life-saving and improved his one-year outcome.

However, there is no universally accepted guideline and imaging modality of choice for the diagnosis and follow-up of this destructive and progressive cardiac disorder [4, 8].

Although STE is a bit more time-consuming, it is an easily performed, non-invasive diagnostic tool for the detection of regional and global left ventricular malfunctions. It has also been useful for the diagnosis of acute myocarditis and to predict the deterioration and the overall event-free survival of such patients [9–11], but its role in EM diagnosis is not clear yet. The case presented here showed the successful determination of the myocardial performance as measured by GLS. Moreover, his STEs showed dynamic changes with appearance and subsequent disappearance of new findings, the reason for which has still remained unanswered. These fascinating STE wax and wanes were seen throughout both admissions and follow-up periods and were detected even when there was no eosinophilia. Their presence, therefore, could be helpful to differentiate EM from other acute life-threatening illnesses such as acute MI or other types of myocarditis. This, of course, needs further investigation.

Another major advantage of STE is that it can detect LV dysfunction long before the occurrence of a drop in LVEF [13]. This, of course, helps earlier diagnosis of EM before an actual drop in LVEF becomes clinically evident or detected by echocardiography.

In addition, longitudinal and circumferential LV strain and strain rate could predict major clinical events in patients with normal or reduced LVEF even after adjustment for confounding variables. Serial STEs showed that areas, which initially seemed to represent scar formation, were subsequently proved to be falsely so and mostly disappeared later on.

Eosinophic myocarditis is a rare clinical entity, and the use of STE in this disorder is only anecdotal. In addition detailed description of their STE findings is even scarcer [14–16].

Although the most common type of LGE involvement is subendocardial in EM subjects, this is not specific for this disease and may occur in other disorders too [17]. In addition, other cardiac segments may get involved as well. Takahara et al. have reported transmural LGE, localized to the anterior wall and global endocardial type has been listed along with other conditions by Franco and colleagues [18].

In this patient, the anteroseptal and anterior walls (or based on segment definitions by Voigt et al. [12], the anterior and anterolateral walls, respectively) were the most frequent sites of involvement, with the middle layer being almost invariably involved (Fig. 3A–D). Further studies are required in this regard to compare the adjunct diagnostic value of STE for EM compared with other diagnostic tools.

The patient had underlying asthma, which could be the source of hyper-eosinophilia and eosinophil deposition in the heart and lung, while withdrawal of steroids and hypersensitivity have also been reported as the source of EM in patients with asthma [19–21]. The increased LV wall thickness in the initial echocardiogram of our patient was most probably because of eosinophilic infiltration, which decreased and gradually normalized by the appropriate treatment (Table 2).

**Table 2:** Showing the serial changes of the left ventricular wall thickness throughout the hospitalizations and follow-ups

Thickness	18.4.2019	23.4.2019	27.4.2019	7.5.2019	3.7.2019	
IVS	12	11	10	9	8	
PFW	12	11	9	9	8	
Thickness	18.7.2019	25.7.2019	19.4.2019	20.11.2019	21.1.2020	25.4.2020
IVS	10	8	8	8	6	8
PFW	10	5	8	8	5	8

*IVS* interventricular septum, *PFW* posterior free wall

An additional issue worth reporting is the value of measuring the left ventricular mechanical dispersion (LVMD) in such patients to predict their arrhythmia vulnerability.

Haugaa and others were the first to report the capability of LVMD to predict the occurance of ventricular arrhythmias and sudden cardiac death in post myocardial infarction patients [22]. More recently, Kawakami and others have shown that for each 10 ms increase in LVMD, there is a significant and independent increment in the ventricular arrhythmic events. They also recommended an optimal cutoff value of 60 ms for LVMD in patients with both ischemic and non-ischemic cardiomyopathies [23].

The mean LVMD value was about 28 ms (18–39 ms) in our patient with no reported or detected major arrhythmias during his hospitalizations and/or several months of out patient follow-ups. Further longer follow-ups and larger number of subjects are however, needed to clearly clarify this important issue in patients with EM.

**Fig. 7:**
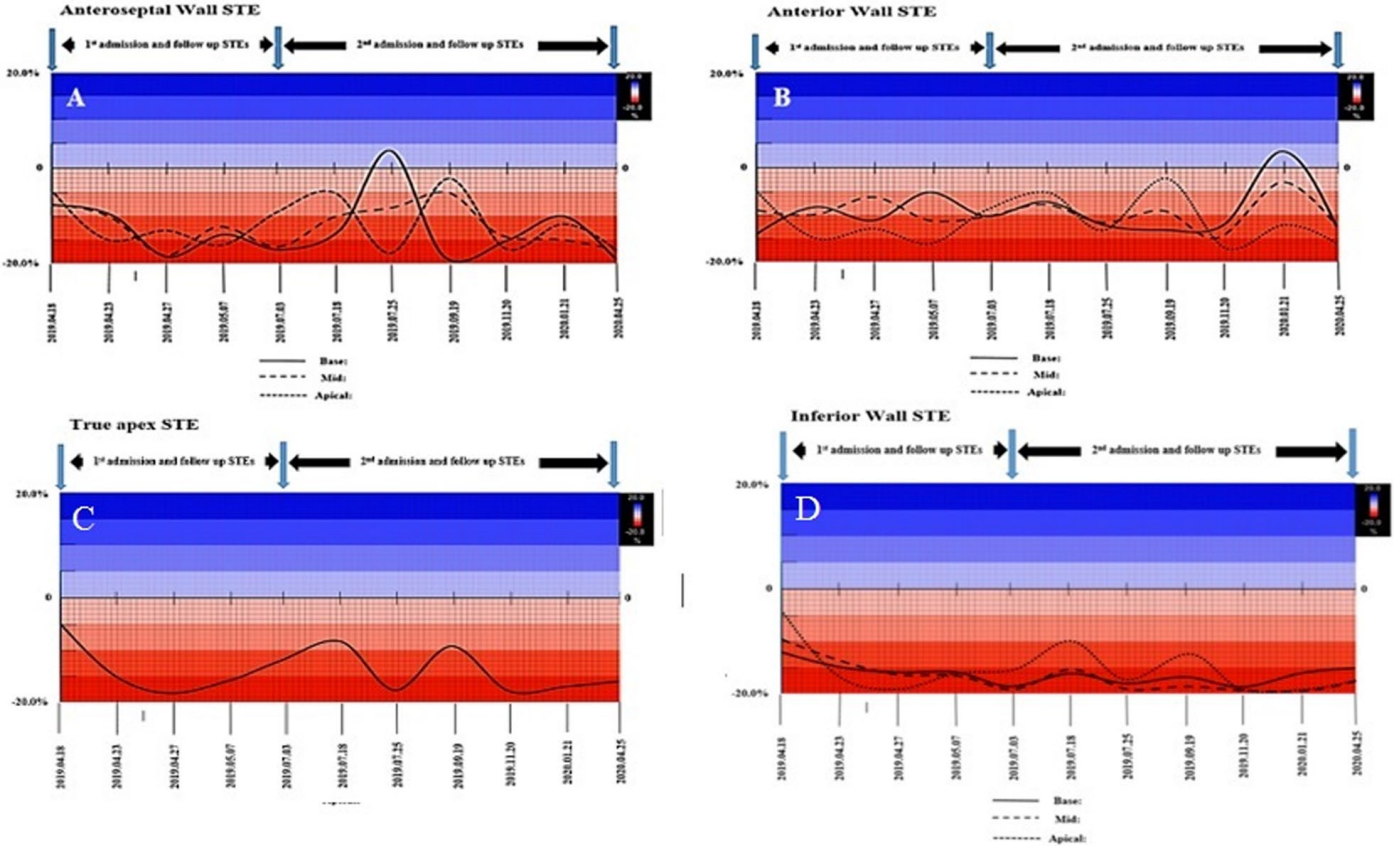
**A**–**D** Speckle tracking echocardiography charts at different cardiac segments. **A** Indicative of the myocardial performance variation during twelve months of follow–up in the different triple segments of the anteroseptal wall. **B** Demonstrating myocardial performance variation during 12 months of follow-up in the different triple segments of the anterior wall. **C** Showing myocardial performance variation during 12 months of follow-up in the true apical segment. **D** Demonstrating myocardial performance variation during 12 months of follow-up in the different triple segments of the inferior wall

In conclusion, the use of STE in this patient proved to have an added value in the evaluation and stratification of the left ventricular function in patients with EM (Fig. 7). The fascinating STE wax and wanes could not only be used for diagnosis of EM but also for its differentiation from other acute life-threatening diseases, such as acute myocardial infarction. This suggests the possible applicability of STE as an adjunct in diagnosis and follow-up of patients with EM, although further studies are required to prove such a phenomenon.

## Data Availability

The data and materials used in this study are available from the corresponding author on reasonable request.

## Abbreviations

EM: Eosinophilic myocarditis
2DE: Two-dimensional echocardiogram
CMR: Cardiovascular magnetic resonance
STE: Speckle tracking echocardiography
LV: Left ventricular
EF: Ejection fraction
RV: Right ventricle
GLS: Global longitudinal strain
LGE: Late gadolinium enhancement

## References

[1] Ramirez GA, Yacoub M-R, Ripa M, Mannina D, Cariddi A, Saporiti N (2018). Eosinophils from physiology to disease: a comprehensive review. Biomed Res Int.

[2] Séguéla P-E, Iriart X, Acar P, Montaudon M, Roudaut R, Thambo J-B (2015). Eosinophilic cardiac disease: molecular, clinical and imaging aspects. Arch Cardiovasc Dis.

[3] Cheung CC, Constantine M, Ahmadi A, Shiau C, Chen LY (2017). Eosinophilic myocarditis. Am J Med Sci.

[4] Sheikh H, Siddiqui M, Uddin SMM, Haq A, Yaqoob U (2018). The clinicopathological profile of eosinophilic myocarditis. Cureus.

[5] Brambatti M, Matassini MV, Adler ED, Klingel K, Camici PG, Ammirati E (2017). Eosinophilic myocarditis: characteristics, treatment, and outcomes. J Am Coll Cardiol.

[6] Balthazar T, Adriaenssens T, Droogne W, Vandenbriele C (2020). Fulminant eosinophilic myocarditis treated with steroids and mechanical unloading: a case report. Eur Heart J Case Rep.

[7] Palecek T, Ganame J, Di Salvo G (2016). Myocardial diseases: current views on etiopathogenesis, diagnostic modalities, and therapeutic options. Biomed Res Int.

[8] Kuchynka P, Palecek T, Masek M, Cerny V, Lambert L, Vitkova I (2016). Current diagnostic and therapeutic aspects of eosinophilic myocarditis. Biomed Res Int.

[9] Løgstrup B, Nielsen J, Kim W, Poulsen S (2016). Myocardial oedema in acute myocarditis detected by echocardiographic 2D myocardial deformation analysis. Eur Heart J Cardiovasc Imaging.

[10] Hsiao J-F, Koshino Y, Bonnichsen CR, Yu Y, Miller FA, Pellikka PA (2013). Speckle tracking echocardiography in acute myocarditis. Int J Cardiovasc Imaging.

[11] Uziębło-Życzkowska B, Mielniczuk M, Ryczek R, Krzesiński P (2020). Myocarditis successfully diagnosed and controlled with speckle tracking echocardiography. Cardiovasc Ultrasound.

[12] Voigt JU, Pedrizzetti G, Lysyansky P, Marwick TH, Houle H, Baumann R (2015). Definitions for a common standard for 2D speckle tracking echocardiography: consensus document of the EACVI/ASE/Industry Task Force to standardize deformation imaging. Eur Heart J Cardiovasc Imaging.

[13] Gunasekaran P, Panaich S, Briasoulis A, Cardozo S, Afonso L (2017). Incremental value of two dimensional speckle tracking echocardiography in the functional assessment and characterization of subclinical left ventricular dysfunction. Curr Cardiol Rev.

[14] Farhat N, Bouhabib M, Joye R, Vallée J-P, Beghetti M (2022). Contribution of imaging modalities to eosinophilic myocarditis diagnosis: a case report. Eur Heart J Case Rep.

[15] Hoppens KR, Alai HR, Surla J, Khokhar HO, Hendel RC (2021). Fulminant eosinophilic myocarditis and VT storm. JACC Case Rep.

[16] Isaza N, Bolen MA, Griffin BP, Popović ZB (2019). Functional changes in acute eosinophilic myocarditis due to chemotherapy with ibrutinib. CASE (Phila).

[17] Franco A, Javidi S, Ruehm SG (2015). Delayed myocardial enhancement in cardiac magnetic resonance imaging. J Radiol Case Rep.

[18] Takahara H, Toba T, Fujimoto D, Izawa Y, Matsumoto K, Tanaka H (2022). Complete resolution of severe secondary mitral regurgitation accompanying eosinophilic myocarditis due to immunosuppressive treatment. J Cardiol Cases.

[19] Dinis P, Teixeira R, Puga L, Lourenço C, Cachulo MC, Gonçalves L (2018). Eosinophilic myocarditis: clinical case and literature review. Arq Bras Cardiol.

[20] Rupani A, Amonkar G, Deshpande J (2010). Eosinophillic myocarditis and coronary arteritis in a fatal case of asthma. Indian J Pathol Microbiol.

[21] Rizkallah J, Desautels A, Malik A, Zieroth S, Jassal D, Hussain F (2013). Eosinophilic myocarditis: two case reports and review of the literature. BMC Res Notes.

[22] Haugaa KH, Grenne BL, Eek CH, Ersbøll M, Valeur N, Svendsen JH (2013). Strain echocardiography improves risk prediction of ventricular arrhythmias after myocardial infarction. JACC Cardiovasc Imaging.

[23] Kawakami H, Nerlekar N, Haugaa KH, Edvardsen T, Marwick TH (2020). Prediction of ventricular arrhythmias with left ventricular mechanical dispersion: a systematic review and meta-analysis. JACC Cardiovasc Imaging.

